# ROCK1 is a potential combinatorial drug target for BRAF mutant melanoma

**DOI:** 10.15252/msb.20145450

**Published:** 2014-12-23

**Authors:** Marjon A Smit, Gianluca Maddalo, Kylie Greig, Linsey M Raaijmakers, Patricia A Possik, Bas van Breukelen, Salvatore Cappadona, Albert JR Heck, AF Maarten Altelaar, Daniel S Peeper

**Affiliations:** 1 Division of Molecular Oncology, The Netherlands Cancer InstituteAmsterdam, The Netherlands; 2 Biomolecular Mass Spectrometry and Proteomics, Bijvoet Center for Biomolecular Research and Utrecht Institute for Pharmaceutical Science, Utrecht UniversityUtrecht, The Netherlands; 3 Netherlands Proteomics CentreUtrecht, The Netherlands; 4 Center for Biomembrane Research, Department of Biochemistry and Biophysics, Stockholm UniversityStockholm, Sweden

**Keywords:** kinome shRNA genomic screening, PLX4720, proteomics, ROCK1

## Abstract

Treatment of BRAF mutant melanomas with specific BRAF inhibitors leads to tumor remission. However, most patients eventually relapse due to drug resistance. Therefore, we designed an integrated strategy using (phospho)proteomic and functional genomic platforms to identify drug targets whose inhibition sensitizes melanoma cells to BRAF inhibition. We found many proteins to be induced upon PLX4720 (BRAF inhibitor) treatment that are known to be involved in BRAF inhibitor resistance, including FOXD3 and ErbB3. Several proteins were down-regulated, including Rnd3, a negative regulator of ROCK1 kinase. For our genomic approach, we performed two parallel shRNA screens using a kinome library to identify genes whose inhibition sensitizes to BRAF or ERK inhibitor treatment. By integrating our functional genomic and (phospho)proteomic data, we identified ROCK1 as a potential drug target for BRAF mutant melanoma. ROCK1 silencing increased melanoma cell elimination when combined with BRAF or ERK inhibitor treatment. Translating this to a preclinical setting, a ROCK inhibitor showed augmented melanoma cell death upon BRAF or ERK inhibition *in vitro*. These data merit exploration of ROCK1 as a target in combination with current BRAF mutant melanoma therapies.

## Introduction

Melanoma is the most aggressive skin cancer and one of the top five most frequent malignancies in the USA (U.S. Cancer Statistics Working Group, 2013). Although treatment of metastatic melanoma has improved recently, no curative therapy is available. Clinically validated driver genes include the mutant oncoproteins BRAF (∼50% of melanomas), c-Kit (∼15%) and NRAS (∼20%) (Gray-Schopfer *et al*, 2007; Hodis *et al*, 2012). The discovery of the common BRAF^V600E^ mutation in melanoma (Davies *et al*, 2002) has resulted in the development of targeted therapies with significant clinical benefits. Until very recently, vemurafenib, a drug targeting BRAF^V600E^, has become the standard of care for patients diagnosed with mutant BRAF metastatic melanoma. However, although this compound or other drugs targeting other components of the MAPK pathway initially reduce tumor burden, eventually all melanomas become resistant and patients succumb to the disease (Flaherty *et al*, 2010; Chapman *et al*, 2011; Wagle *et al*, 2011).

Drug resistance in this setting is caused by a plethora of mechanisms, both MAP kinase dependent and independent, making it virtually impossible to design a single effective targeted therapy from which all patients would benefit. For example, several mutations located in the MAPK pathway have been detected in vemurafenib-resistant cell lines or patient tumors, including activating mutations in MEK1 (Wagle *et al*, 2011; Trunzer *et al*, 2013), MEK2 (Wagle *et al*, 2014) and in NRAS (Nazarian *et al*, 2010). In addition, long-term treatment with BRAF inhibitors has been shown to induce switching between RAF isoforms and amplification of BRAF^V600E^ or expression of an alternative, 61-kDa RAF splice variant lacking the RAS-binding domain, causing constitutive activation of the MAP kinase pathway (Villanueva *et al*, 2010; Poulikakos *et al*, 2011; Shi *et al*, 2012). Furthermore, overexpression of CRAF is associated with BRAF resistance (Montagut *et al*, 2008). Other mechanisms, not involving the MAPK pathway, have been found as well, including up-regulation of IGF-1R (Villanueva *et al*, 2010), PDGFRβ (Nazarian *et al*, 2010), FOXD3 (Basile *et al*, 2012), EGFR (Girotti *et al*, 2013; Sun *et al*, 2014) or FGFR3 (Yadav *et al*, 2012) signaling. Overexpression of COT (Johannessen *et al*, 2010), Cyclin D1 (Smalley *et al*, 2008) and AEBP1 (Hu *et al*, 2013), amplification of MET and CTNNB1 (Vergani *et al*, 2011) and loss of NF1 (Whittaker *et al*, 2013) can also confer resistance to vemurafenib. The micro-environment can play a role in resistance, as it was found that up-regulation of HGF by the surrounding stromal cells occurs during resistance (Straussman *et al*, 2012). Lastly, BRAF inhibitor-resistant tumors have increased levels of autophagy (Ma *et al*, 2014).

Since many resistance mechanisms are MAPK pathway dependent, clinical trials in which melanoma patients are treated simultaneously with BRAF and MEK inhibitor are ongoing (Flaherty *et al*, 2012). Although the clinical responses may improve as a result, resistance continues to be a major problem (Shi *et al*, 2014; Wagle *et al*, 2014). Therefore, it is important to identify novel therapeutic targets that can be used in the treatment of melanoma patients in combination with, or instead of, existing therapies. As vemurafenib is the best-characterized drug for melanoma treatment, we pursued a multi-angle approach, utilizing an integrated and unbiased proteomic and genetic screening platform to identify targets whose inhibition would increase the toxicity of vemurafenib toward melanoma cells.

## Results

### Mass spectrometry (MS) analysis identifies induction of proteins involved in melanoma survival

**Figure 1:**
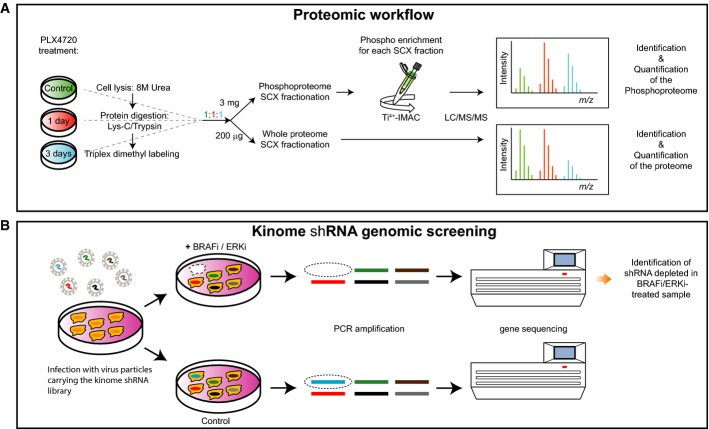
Proteomic and genomic workflows Cell lysates from control samples and samples derived from 1 and 3 days after PLX4720 treatment were digested with Lys-C/Trypsin, labeled by triplex dimethyl approach and mixed in 1:1:1 ratios. For protein expression analysis, 200 μg of digested lysate was fractionated by SCX and each fraction was analyzed by LC/MS/MS to determine the relative protein expression levels for every time point compared to the control. For the phosphoproteome, 3 mg of digested lysate was fractionated by SCX and each fraction was enriched for phosphopeptides by Ti^4+^-IMAC prior to LC/MS/MS analysis.

To gain insight into the molecular mechanisms and signaling pathways underlying normal drug response and thereby screen for novel targets for drug sensitization, we combined (phospho)proteomic analysis and an shRNA library function-based approach. We used a low-passage human melanoma cell line, 04.01, which is sensitive to treatment with PLX4720, a preclinical vemurafenib analog. For the MS-based proteomic approach, we performed a time course experiment, monitoring the changes of the proteome and phosphoproteome after 1 and 3 days of treatment with an IC_50_ dose of PLX4720 (Fig1A). Alongside, we performed two shRNA screens on the same cell line to identify shRNAs that sensitize melanoma cells to targeted inhibition of either BRAF or one of its critical effectors, ERK (Fig1B; described in detail below).

Melanoma cells were transduced with a lentiviral kinome library, containing ˜4,000 shRNAs targeting ˜500 kinases. Cells were treated either with DMSO (control) or with BRAFi or ERKi. Genomic DNA was isolated, and hairpins were amplified by PCR. Using deep sequencing, the hairpins that specifically dropped out in the treated sample were identified. In this case, the absence of the blue bar in deep sequencing indicates schematically a synthetic lethal effect of the shRNA and BRAFi/ERKi.

**Figure 2:**
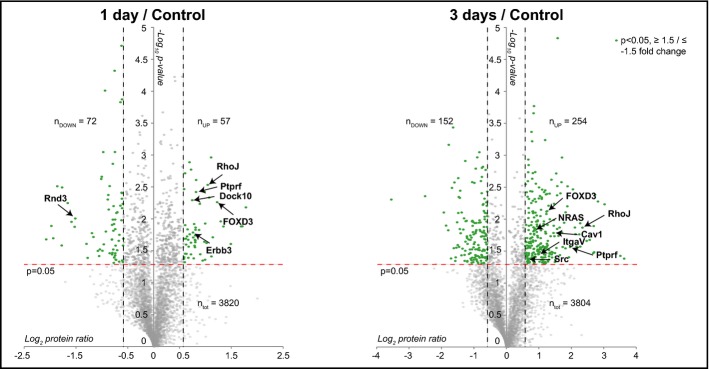
MS analysis identifies proteins potentially contributing to melanoma survival Volcano plots of protein expression levels at 1 day (left panel) and 3 days (right panel) compared to control sample. Statistically significant entries with a *P*-value < 0.05 and fold change ≥ 1.5 and ≤ −1.5 are labeled in green (one-sample *t*-test against 0). For simplicity, only some proteins have been tagged. n_tot_, n_UP_ and n_DOWN_ indicate the total number of proteins quantified in the three biological replicates and the number of statistically significant proteins that are up-regulated and down-regulated in the three biological replicates, respectively.

For the proteome and phosphoproteome analyses, cells were lysed, digested by Lys-C/Trypsin, labeled using the dimethyl approach (Boersema *et al*, 2009), mixed 1:1:1 and fractionated by strong cation exchange (SCX) (Fig1A). For the phosphoproteome analysis, SCX fractions were subsequently subjected to phosphopeptide enrichment by Ti^4+^-IMAC (Zhou *et al*, 2013) and analyzed by LC/MS/MS on an LTQ-Orbitrap Velos or Elite using a data-dependent decision tree MS/MS method (ETD-IT or HCD). Here, the most suitable fragmentation technique is automatically selected (according to the charge and *m*/*z*) to enhance the number of phosphopeptide identifications (Frese *et al*, 2011). For the whole proteome analysis, the SCX fractions were analyzed on an Orbitrap Velos, Elite or Q-Exactive (Fig1A). Overall, ∼5,700 proteins and ∼11,500 phosphosites (80% with a location probability ≥ 75%) were identified from the three biological replicates with a protein and peptide FDR ≤ 1%. At the protein level, we quantified ∼3,800 proteins over all conditions and found 129, 406 and 313 proteins regulated significantly at 1 day/control, 3 days/control and 3 days/1 day, respectively, after performing a statistical assessment (*P* < 0.05), and choosing an arbitrary fold change cutoff of 1.5, corresponding to a total of 588 unique regulated proteins (Fig2; Supplementary Table S1). Network analysis of the significantly changing proteins using Reactome as plugin in Cytoscape (Haw *et al*, 2011) revealed a high number of regulated protein–protein interactions at 3 days/control and a substantial up-regulation of membrane proteins at 3 days/1 day (Supplementary Fig S1). A gene ontology (GO) analysis using Panther (http://www.pantherdb.org) (Mi,^2013^) was performed on the proteins whose expression changed significantly. The most profound changes in protein expression levels were observed at 3 days, when receptors became particularly over-represented (Supplementary Fig S2; Supplementary Table S2). A GO slim analysis of the proteome and phosphoproteome data using BiNGO as Cytoscape plugin (Maere, 2005) revealed enrichment for cytoskeleton organization (Supplementary Fig S3; Supplementary Table S3).

**Figure 3:**
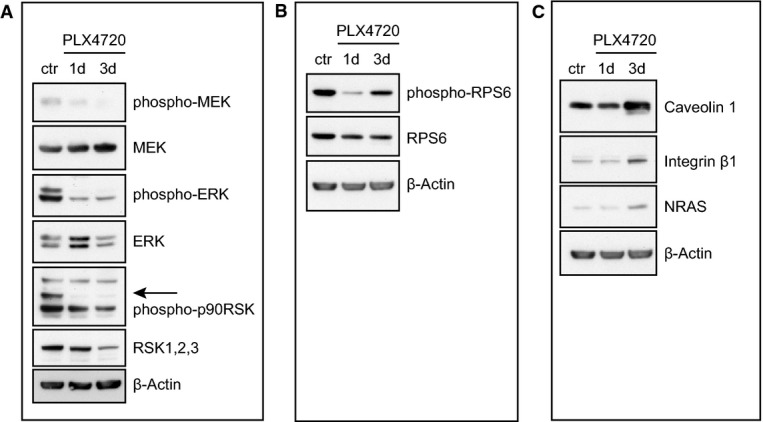
Western blot analysis confirms deactivation of the MAPK pathway and MS-based phosphosite and protein quantification Cells were plated and treated the next day with either DMSO as a control (ctr) or 0.5 μM PLX4720 for 1 (1d) and 3 days (3d) and analyzed for proteins as indicated. β-actin serves as a loading control. MEK, ERK and p90RSK phosphorylation levels.

As expected, exposure to PLX4720 led to down-regulation of the phosphorylation state of kinases within the MAPK pathway: phospho-MEK, phospho-ERK1/2 and phospho-p90RSK (Fig3A). Indeed also in our mass spectrometry analysis, we observed strong down-regulation of the phosphorylation states of ERK1 (at 1 and 3 days) and ERK2 (at 3 days; Supplementary Table S1), which indicate a responsive state of the employed cell line. Moreover, the phosphorylation state of RPS6 was significantly down-regulated especially at 1 day, indicative of an inactive state of the mTORC1 pathway (Fig3B, Supplementary Table S1) (Roux *et al*, 2007).

RPS6 phosphorylation levels.

Expression levels of several MS-quantified proteins.

Regarding the protein expression analysis, a number of proteins were differentially regulated (Supplementary Table S1); for simplicity, only a select set is depicted in Fig2. This unbiased approach enabled us to observe also a number of changes upon PLX4720 treatment previously described in relation to drug resistance. For example, we detected up-regulation of the transcription factor FOXD3 at 1 and 3 days (Fig2) and one of its transcriptional targets, the receptor protein kinase Erbb3, at 1 day (Fig2, left panel). This is consistent with previous findings, where FOXD3 has been found to confer resistance to PLX4720-induced cell death by up-regulation of Erbb3 (Basile *et al*, 2012; Abel *et al*, 2013). Moreover, we found several proteins that were changed upon PLX4720 treatment that play a role in lipid rafts. First, at 3 days, the lipid raft-associated proteins, caveolin 1 and 2, and raftlin (Cav1, Cav2, Rftn1), were consistently up-regulated (Fig2, right panel; Supplementary Table S1). Interestingly, Cav1 has been reported to promote growth and invasion of melanoma cells (Felicetti *et al*, 2009). Second, at 3 days, we observed the up-regulation of integrin alfa V (ItgaV) (Fig2, right panel), whose expression has been reported to be positively regulated by Cav1 (Arpaia *et al*, 2012). Indeed, we found several other integrins to be up-regulated such as Itga4, Itga1, Itga2, Itgb5 and Itgb1 (Supplementary Table S1). Third, Ptprf (or Lar) was up-regulated in time (Fig2), and this phosphatase has been reported to be localized in lipid rafts (Caselli *et al*, 2002). Fourth, we detected up-regulation of Src at 3 days (Fig2, right panel). Src membrane family proteins (Src, Fyn and Yes) are recruited for signal transduction in the cholesterol-rich lipid raft membrane structures (Liang *et al*, 2001). Interestingly, Src kinase has been implicated in mechanisms of resistance to vemurafenib (Vergani *et al*, 2011; Girotti *et al*, 2013). The biological relevance of the lipid rafts in melanoma has been further suggested by the recent work of Zanfardino *et al* (2013) who demonstrated that simvastatin, a compound that can block cholesterol synthesis, reduces tumor growth in a melanoma xenograft. We also observed other proteins involved in PLX4720 resistance. At 3 days, we detected an increase of NRAS (Fig2, right panel), whose up-regulation is involved in vemurafenib resistance (Nazarian *et al*, 2010). At 1 and 3 days, we also observed a significant up-regulation of RhoJ, which has been associated with melanoma invasion and chemoresistance (Ho *et al*, 2012, 2013) (Fig2). In an independent experiment, we validated some of the identified proteins by Western blot analysis, which confirmed the upregulation of integrin beta 1, caveolin 1 and NRAS after 3 days (Fig3C). Overall, we conclude from our MS-based proteomics time course experiment that melanoma cells upon treatment with PLX4720 induce expression of multiple proteins that have been previously reported to contribute to vemurafenib resistance, as well as several novel proteins (Supplementary Table S1).

### Parallel function-based shRNA screens identify factors contributing to melanoma cell survival

**Figure 4:**
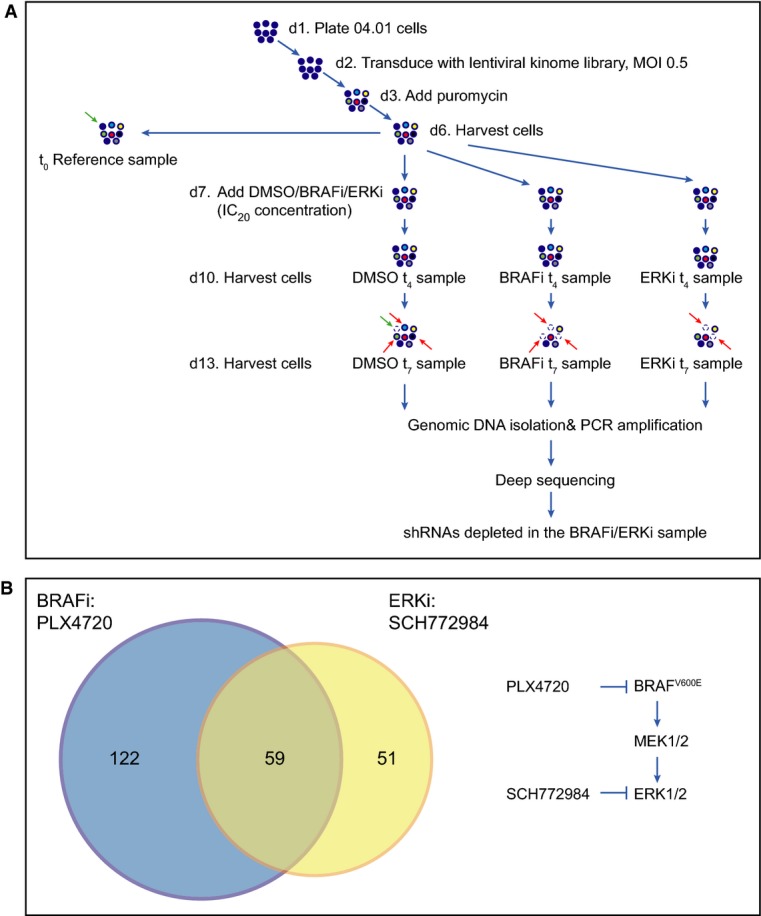
RNAi screens identify short hairpins sensitizing to BRAFi or ERKi treatment 04.01 melanoma cells were transduced with the kinome shRNA library (which was divided in four pools each containing ˜1,000 hairpins) with a multiplicity of infection (MOI) of 0.5. After selection with puromycin, cell pools were divided into four samples: one as a reference control, one was treated with DMSO as a control, one was treated with 0.15 μM PLX4720 and one was treated with 0.015 μM SCH772984. After 4 and 7 days of treatment, cells were harvested. Genomic DNA was isolated and deep sequenced. shRNAs that dropped out in the treated sample are highlighted by dashed empty circles and indicated by red arrows compared to the control. Hairpins that dropped out in the untreated sample compared to the reference sample are highlighted by dashed empty circles and indicated by green arrows.

Parallel to the proteomic analysis, we set out to perform an unbiased and function-based genomic screen to identify potential drug target(s) that can be used in combination with BRAF inhibitors. Specifically, we performed a sensitizer screen for PLX4720 with a lentiviral shRNA library containing ∼4,000 shRNAs targeting ∼500 different kinases. We transduced the human melanoma cell line 04.01 with a multiplicity of infection (MOI) of 0.5 to limit the likelihood of multiple integrations per cell, avoiding competition for the RNAi silencing machinery. After pharmacologic selection, cells were treated for 1 week with either DMSO (control) or low-concentration PLX4720 (IC_20_). Next-generation sequencing was used to identify hairpins selectively depleted in the treated sample compared to the control, with the aim to identify drug enhancers (FigA). As already described in the introduction, many resistance mechanisms to BRAF inhibition involve re-activation of ERK. Also ERK inhibitors are currently being developed to treat melanoma (Morris *et al*, 2013). Therefore, we performed an ERK inhibitor (SCH772984) sensitization screen in parallel. For both screens, we selected the hairpins that were significantly depleted (*P* < 0.05) in the treated sample by at least 1/3 after 1 week of treatment. We excluded hairpins that were enriched after 4 days of treatment, to minimize the number of false-positive hits. We compared the identified hairpins from both screens (PLX4720 and SCH772984) and found an overlap of 59 (Fig4B, Supplementary Table S4). Selecting the genes that were silenced by at least two hairpins in both screens (to exclude potential off-target effects and to increase the likelihood that the hits functionally interacted with the BRAF/MEK/ERK pathway) yielded five potential hits: AAK1, PLK4, IGF1R, MET and ROCK1.

Venn diagram depicting the overlap of identified hairpins in the PLX4720 and SCH772984 sensitizer screens. On the right, the BRAF pathway and the two inhibitors used are depicted.

### Integration of proteomic and functional genomic analyses reveals regulation of ROCK signaling upon PLX4720 treatment

To select the hit from our sensitizer screens with the highest potential as drug companion target, we integrated our functional screening results with the proteomic data. Interestingly, we found several regulators of ROCK1 in our proteomic dataset. First, we detected significant down-regulation of Rnd3 upon PLX4720 treatment (Fig2, left panel). Rnd3 is a negative regulator of ROCK1 activity (Riento *et al*, 2003; Belgiovine *et al*, 2010) and fitting with our observation, Rnd3 is induced by RAF transformation in epithelial cells (Hansen *et al*, 2000). Furthermore, in melanoma cells that persist after BRAF inhibition, Rnd3 restoration decreased cell invasion (Klein & Higgins, 2011). Interestingly, FOXD3, also identified in our MS analysis and an established player in PLX4720 resistance (see above), is linked to ROCK1, as it down-regulates Rnd3 in melanoma cells (Katiyar & Aplin, 2011). Notably, overstimulation of the MAPK pathway (resulting from transformation by RAS^V12^) has been reported to negatively regulate ROCK1 expression in fibroblasts (Sahai *et al*, 2001; Pawlak & Helfman, 2002). ROCK1 is known to regulate the switch toward amoeboid motility (Sanz-Moreno & Marshall, 2010; Sanz-Moreno *et al*, 2011), and this round morphology has been associated to enhanced aggressiveness and motility of melanoma cells (Ramgolam *et al*, 2011). Second, after 24 h of PLX4720 treatment, we observed up-regulation of Dock10 in our proteomic data (Fig2, left panel), a guanine exchange factor (GEF) of Cdc42, which is required for amoeboid movement (Gadea *et al*, 2008). Third, as mentioned already, we found a number of integrins, including Itgb1, up-regulated upon PLX4720 treatment. Integrins are involved in cell adhesion/movement, and Itgb1 has been shown to activate the RhoA-ROCK pathway in colon carcinoma cells (Vial *et al*, 2003). Taking the data of both shRNA screens together, and integrating them with our proteomic analysis results, yielded the serine threonine kinase ROCK1 as an interesting and potential drug co-target in melanoma therapy.

### ROCK1 silencing sensitizes melanoma cells to BRAF and ERK inhibition

**Figure 5:**
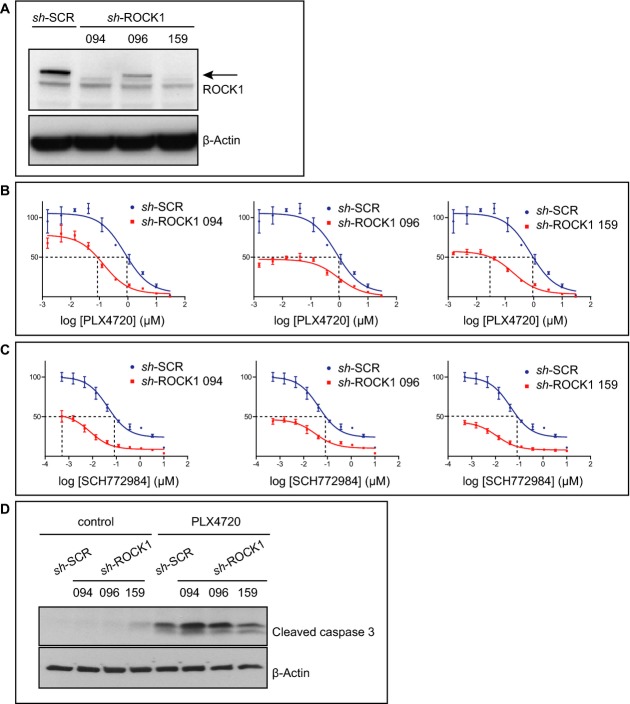
ROCK1 silencing sensitizes melanoma cells to BRAF or ERK inhibition A Cells were transduced with sh-SCR(ambled) as a control or with one of the three different shRNAs against ROCK1 and analyzed for ROCK1 levels by Western blot analysis. β-actin served as a loading control.

Four and two different hairpins were depleted for ROCK1 in the PLX4720 and SCH772984 sensitizer screens, respectively (Table1). We next validated this in an independent experiment. Silencing of ROCK1 with three different hairpins resulted in decreased levels of ROCK1 (Fig5A). Confirming our screen results, silencing of ROCK1 resulted in fewer viable cells upon treatment with either PLX4720 or SCH772984 as analyzed by dose response curves (Fig5B and C). To determine whether, indeed, ROCK1 silencing renders the cells more sensitive to death upon PLX4720 treatment, we analyzed the levels of the pro-apoptotic protein-cleaved caspase 3 in treated cells. Treatment of PLX4720 showed increased cleaved caspase 3 levels, consistent with dose response analysis. Although silencing ROCK1 had no effect on cleaved caspase 3 levels under normal conditions, upon treatment with PLX4720, cells with silenced ROCK1 had a further increase in cleaved caspase 3 levels (Fig5D).

**Table 1:** Fold change of the hairpins identified in the PLX4720 and SCH772984 sensitizer screen.

ROCK1			
PLX4720		SCH772984	
Hairpin	Fold change	Hairpin	Fold change
94	0.46	94	0.62
96	0.42		
159	0.52	159	0.6
161	0.54		

B, C Cells described in (A) were treated with a dilution series of PLX4720 (B) or SCH772984 (C) for 3 days. Cell viability was determined with a cell titer blue assay. The *y*-axis represents the percentage of living cells, normalized to cells expressing sh-SCR. Error bars represent standard error of the mean of one representative experiment done in triplicate. Dashed lines represent the change in IC_50_.

D Cells described in (A) were plated and treated with 0.15 μM PLX4720 on the next day. After 3 days, cells were harvested (apoptotic cells in the supernatant were included in the analysis) and analyzed by Western blot. β-actin served as a loading control.

**Figure 6:**
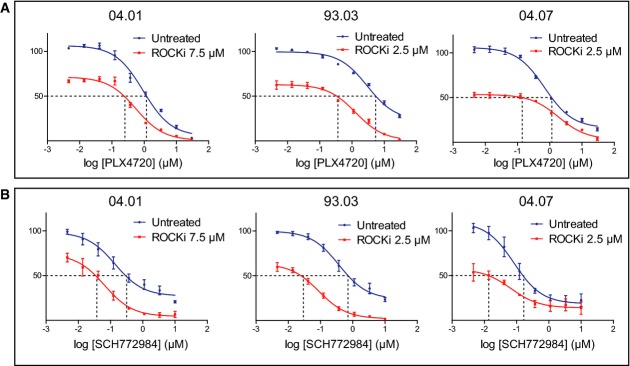
Targeted ROCK inhibition increases the toxicity of inhibitors of the MAPK pathway Three independent melanoma cell lines (04.01, 93.03 and 04.07) were treated with dilution series of PLX4720 either alone or in combination with the ROCK inhibitor GSK269962A. After 3 days, cell viability was determined by a cell titer blue assay and represented in the *y*-axis.
Cells were treated same as in (A), but with a dilution series of the ERK inhibitor SCH772984.

To begin to translate these findings to a more clinically relevant setting, we turned to pharmacologic inhibition of ROCK. The ROCK inhibitor fasudil is being used for treatment of cerebral vasospasms (Olson, 2008). We examined whether inhibition of ROCK1 with a pharmacological inhibitor could have an additive effect on BRAF or ERK inhibition. For this purpose, we used GSK269962A, an inhibitor targeting ROCK1 and ROCK2, which has been shown to have vasodilatory effects in rats (Doe *et al*, 2007) and which is more specific than fasudil. We treated six different low-passage melanoma cell lines with a dilution series of PLX4720 or SCH772984 with or without a fixed concentration of the ROCK inhibitor. Indeed, addition of the ROCK inhibitor profoundly enhanced the effect of either PLX4720 or SCH772984 to induce melanoma cell death (Fig6A and B, Supplementary Fig S4). Furthermore, in the 93.03 and A375 melanoma cell lines, addition of ROCK inhibitor eliminated the population of residual cells that survived at the highest PLX4720 or SCH772984 concentration. This suggests that combined treatment with a ROCK inhibitor and a BRAF inhibitor may be beneficial for patients with BRAF mutant melanoma.

Data information: Errors bars represent standard error of the mean of three independent experiments. Dashed lines represent the change in IC_50_.

## Discussion

**Figure 7:**
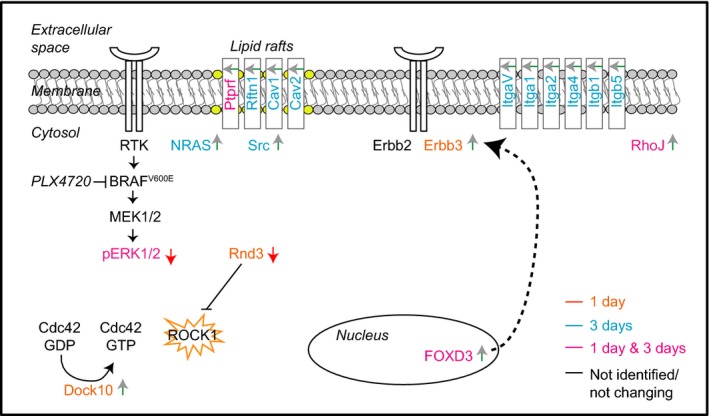
Cartoon representing selected pathways detected by (phospho)proteomic analysis Up-regulated or down-regulated proteins/phosphosites are indicated by a green or red arrow, respectively. Proteins regulated at 1 day/control only, 3 days/control only and entries regulated in both time points are labeled in orange, blue and pink, respectively. Proteins that have not been identified or whose expression levels are not changing are labeled in black. ROCK1 has been highlighted by an orange star as potential co-target in combination with PLX4720 therapy according to our integrated proteomic and genomic approach.

Here, we applied an unbiased multi-angle approach to discover new potential targets that render melanoma more sensitive to clinically relevant inhibitors of the BRAF pathway, particularly those targeting BRAF and ERK. As a model system, we used a panel of BRAF mutant human melanoma cell lines sensitive to PLX4720 treatment. Interestingly, although we used a sensitive cell line in our proteomic platform, we detected elevated expression of several proteins that have previously been reported to be involved in resistance. They include NRAS, Erbb3 and Src, and the transcription factor FOXD3 (Fig7). These observations raise the interesting possibility that melanoma cells activate multiple different pathways involved in resistance already very early on, as soon as they are exposed to targeted inhibition of driver oncoproteins. Our data provide a resource for future studies aiming to resolve the mechanism by which certain responses are selected in pathways leading to drug resistance and survival of the melanoma cells. Indeed, as we show here, the impairment of one of these drug-regulated pathways can be effective in improving current targeted melanoma therapies.

In our function-based and unbiased genomic approach, we employed two screens to identify factors whose targeted silencing sensitizes melanoma cells to BRAF pathway inhibition. Consistent with the fact that the inhibitors PLX4720 and SCH772984 target the same pathway, there are several overlapping hairpins between the two screens, increasing the robustness of this approach. Because of this overlap, we were able to stringently select for factors that are useful as targets in combination with inhibition of the BRAF/MAPK pathway in melanoma. The strength of this approach is illustrated by the finding that two out of the five hits, IGFR and MET, have already been implicated in resistance to BRAF inhibition (Villanueva *et al*, 2010; Vergani *et al*, 2011; Straussman *et al*, 2012).

By combining our genomic data with (phospho)proteomic analyses, we were able to identify a new target, ROCK1, whose inhibition rendered melanoma cells much more sensitive to BRAF/MAPK inhibition. While PLX4720 treatment influenced ROCK1 signaling, silencing of ROCK1 as well as the use of a pharmacologic ROCK inhibitor significantly increased the elimination of melanoma cells by PLX4720 and SCH772984 treatments. The use of ROCK1 as a potential target in cancer therapy has been suggested previously (Rath & Olson, 2012). Single ROCK1 inhibition reduces tumor outgrowth (Patel *et al*, 2012) and bone metastasis in breast cancer (Liu *et al*, 2009), while in prostate cancer, ROCK1 silencing reduces tumor growth (Zhang *et al*, 2013). However, ROCK inhibitors have not been extensively explored in melanoma. The only studies that show an effect of ROCK inhibition on melanoma growth have been performed on mouse cells (Nakajima *et al*, 2003; Routhier *et al*, 2010). To our knowledge, the use of ROCK inhibitors has never been reported in a combinatorial approach with the exception of one study combining ROCK inhibition and taxol on murine melanoma cells (Nakajima *et al*, 2003). Our results are consistent with and extend these findings, meriting further exploration of the use of ROCK inhibition in melanoma treatment.

Because resistance to single drug treatment is seen almost invariably in melanoma and other cancer types, combination treatments need to be developed and used as early as possible to keep the tumor burden limited at early stages. Indeed, the combination of vemurafenib with the MEK inhibitor trametinib is tested in a clinical trial (Flaherty *et al*, 2012). However, resistance still occurs upon this treatment, for example, owing to mutations in MEK (Wagle *et al*, 2014). Furthermore, also the subsequent treatment of MEK and BRAF inhibitors has been tested, but the differences compared to combined treatment remain small (Goldinger *et al*, 2014). A combination of a BRAF and PI3K inhibitor effectively eliminates BRAF inhibitor-resistant melanoma cell line clones (Greger *et al*, 2012; Vredeveld *et al*, 2012); currently, a clinical trial using vemurafenib and BKM120, a PI3K inhibitor, is ongoing (ClinicalTrials.gov). Since resistance is caused by a multitude of events, targeting two or even more nodes simultaneously is likely to be beneficial, as many have suggested (Greger *et al*, 2012; Vredeveld *et al*, 2012; Kaplon *et al*, 2013; Villanueva *et al*, 2013). Unlike vemurafenib, ERK inhibitors are not yet beyond clinical trials for the treatment of melanoma. So far, it has been reported that ERK inhibitors have anti-tumor effects in melanoma xenografts and that ERK inhibition can overcome MEK and BRAF inhibitor resistance (Morris *et al*, 2013). Furthermore, combination of ERK and PI3K/mTOR inhibition promotes cell death in resistant melanoma cells (Carlino *et al*, 2014). Our results warrant exploration of combinations of BRAF/ROCK inhibition or ERK/ROCK inhibition in further studies for the treatment of BRAF mutant melanoma.

## Materials and Methods

### Constructs and inhibitors

The shRNA kinome library contains hairpins targeting around 500 kinases. This library was assembled from The RNAi Consortium (TRC) human genomewide shRNA collection (TRC-Hs1.0) (OpenBiosystems) in four different pools, each containing around 1,000 hairpins. pLKO-SCR, pLKO-shROCK1 #094 (ATTTACCTCTTGTTCTAACCG), #096 (TATGTCCAATACCATAGATGG) and #159 (TACTTTGTGTTCATTTACCTC) were from the TRC-Hs1.0 library (OpenBiosystems). Inhibitors used were PLX4720 (Selleck), SCH772984 (Merck, via a MTA), GSK269962A (Axon Medchem) and metabolic poison phenyl arsine oxide (PAO) (Sigma).

### Cell culture and viral transductions

BRAF^V600E^ melanoma cells 04.01, 04.07, 93.03 and 00.08 were from Leiden University Medical Center, and the cell line identity was verified with STR profiling (PowerPlex 16 HS, Promega). These melanoma cells, mel888, A375 and HEK293T, were cultured in Dulbecco's modified Eagle's medium (DMEM) (Gibco) supplemented with 9% fetal calf serum (Sigma) and penicillin + streptomycin. For lentiviral transductions, HEK293T was transfected with 8 μg plasmid in medium containing 25 mM chloroquine, using the helper plasmids pMDLglpRRE, pHCMV-G and pRSVrev, and refreshed after 6 and 24 h. After 48 h, virus supernatant was harvested and diluted for transduction. 04.01 melanoma cells were transduced with pLKO-sh-SCR or pLKO-sh-ROCK1 in the presence of 4 μg/ml polybrene and selected with 1.0 μg/ml puromycin. Four days after transduction, cells were set up for further experiments.

### Kinome shRNA screen

04.01 melanoma cells were transduced with the four different pools of the shRNA kinome library at an MOI of 0.5. The next day, 1.0 μg/ml puromycin was added to the cells. Following 3 days of puromycin selection, cells were divided into three groups: a reference control, a DMSO-treated sample and a sample treated with the IC_20_ concentration of either PLX4720 (0.15 μM, for the PLX4720 screen) or SCH772984 (0.015 μM, for the ERKi screen). Cells were harvested after 4 and 7 days of treatment with the respective inhibitors. Genomic DNA was isolated, and shRNAs were amplified by PCR using a FOR primer incorporating the Illumina sequencing primer and a 6-bp index to allow discrimination of different samples (5′-ACACTCTTTCCCTACACGACGCTCTTCCGATCT_INDEX_CTTGTGGAAAGGACGAAACACCGG-3′), and a REV primer incorporating a P7 arm (5′-CAAGCAGAAGACGGCATACGAGATTTCTTTCCCCTGCACTGTACCC-3′). A second round of PCR was then performed using a FOR primer incorporating a P5 arm (5′-AATGATACGGCGACCACCGAGATCTACACTCTTTCCCTACACGACGCTCTTCCGATCT-3′) and the P7 REV primer. The PCR products were purified and analyzed by next-generation sequencing (Illumina HiSeq2000). The screens were performed independently three times, and results were analyzed with DESeq version 1.4.1 from R/Bioconductor (Gentleman *et al*, 2004; Anders & Huber, 2010). Hairpins were selected by comparing treated samples to DMSO-treated samples at day 7 (fold change 0.66, *P* < 0.05) and treated samples to DMSO-treated samples at day four (fold change < 1). Finally, hairpins from the two different screens (the PLX4720 and ERKi screen) were compared, and genes that were targeted by at least two hairpins in both screens were identified as hits for further analysis.

### Dose response curves, drug treatments and mass spectrometry analysis

Equal cell numbers were plated on 96 wells, and the next day, cells were treated with different concentrations of indicated inhibitor(s). After 3 days, cell viability was determined with a cell titer blue assay (Promega), and fluorescence was quantified by a TECAN Infinite M200 scanner. Values were normalized to a negative control (no treatment, set at 100%) and a positive control for killing (PAO, set at 0%). For (phospho)proteomic analysis, 04.01 cells were plated on 10-cm plate, and the next day, cells were treated with vehicle or 0.5 μM PLX4720. Cells were harvested the same day (control vehicle treated cells), after 24 h and after 3 days. Cells in the supernatant were not included in the analysis. For drug treatments for Western blot analysis, 04.01 melanoma cells expressing sh-SCR or sh-ROCK1 were treated with 0.15 μM PLX4720 for 3 days. Cells in the supernatant were included in the Western blot analysis.

### Western blot analysis

Samples were lysed in RIPA (50 mM TRIS pH 8.0, 150 mM NaCl, 1% Nonidet P40, 0.5% sodium deoxycholate, 0.1% SDS) in the presence of a protease inhibitor cocktail (Roche) and phosphatase inhibitors (1 mM sodium pyrophosphate, 2 mM sodium fluoride, 10 mM β-glycerophosphate, 2 mM orthovanadate). Protein concentration was determined using a Bradford assay (Bio-Rad). Immunoblot analysis was performed using standard techniques on 4–12% bis-tris precast gels (NuPAGE). Proteins were transferred on nitrocellulose membranes (Millipore). Primary antibodies were ROCK1(BD Transduction Laboratories), β-actin (AC74, Sigma), cleaved caspase 3 (Asp175), caveolin 1, phopho-MAPK (Thr202/Tyr204), MAPK, phopho-MEK (41G9), MEK (L38C12), phospho-p90RSK (Thr359/Ser363), RSK (against RSK1, 2, and 3) phospho-RPS6 (Ser235/236), RPS6 (all Cell Signaling), integrin β1 (Bio-Connect) and NRAS (F155, Santa Cruz). Protein detection was performed using ECL agent (Amersham), and developed films were scanned on an Epson Perfection 4990 Photo scanner.

### Sample preparation for mass spectrometry analysis

The cellular pellets of control sample and cells exposed to 1 and 3 days of PLX4720 treatment were harvested and resuspended in lysis buffer [8 M Urea in 50 mM triethyl ammonium bicarbonate, pH 8.5, 1 mM sodium orthovanadate, 1 tablet of Complete mini EDTA-free mixture (Roche Applied Science) and one tablet of PhosSTOP phosphatase inhibitor mixture per 10 ml of lysis buffer (Roche Applied Science)]. Cells were then lysed by 10 rapid passages through a 23-gauge hypodermic syringe needle and by sonication on ice. After centrifugation (20,000 × *g* 30 min at 4°C), the protein concentration was determined by Bradford assay (Pierce). Proteins were reduced with 2 mM DTT at 56°C for 25 min, alkylated with 4 mM iodoacetamide at room temperature for 30 min in the dark and reduced again with 2 mM DTT at room temperature to prevent over-alkylation. A first enzymatic digestion step was performed in 8 M urea lysis buffer using Lys-C at 37°C for 4 h (enzyme/substrate ratio 1:50). The sample was diluted four times with 50 mM triethyl ammonium bicarbonate pH 8.5 and digested overnight at 37°C with Trypsin (enzyme/substrate ratio 1:50). Finally, the digestion was quenched with 5% formic acid. The resulting peptides were chemically labeled using stable isotope dimethyl labeling as described before (Boersema *et al*, 2009). The protein digests from the control sample, 1 day and 3 days of PLX4720 treatment were labeled as “Light” (L), “Medium” (M) and “Heavy” (H), respectively. An aliquot of each label was measured on a regular LC-MS/MS run, and samples were mixed 1:1:1 (L:M:H) based on their peptide intensities and dried down. The procedure was repeated in three biological replicates.

### Protein expression levels analysis

The labeled peptides were reconstituted in 10% formic acid prior to fractionation using strong cation exchange (SCX) as described previously (Helbig *et al*, 2010) for the protein expression levels analysis. The SCX system consisted of an Agilent 1100 HPLC system (Agilent Technologies, Waldbronn, Germany) with two C_18_ Opti-Lynx (Optimized Technologies, OR) trapping cartridges and a polysulfoethyl A SCX column (PolyLC, Columbia, MD; 200 mm × 2.1 mm inner diameter, 5 μm, 200-A). The peptides were dissolved in 10% FA and loaded onto the trap columns at 100 μl/min and subsequently eluted onto the SCX column with 80% acetonitrile (ACN; Biosolve, the Netherlands) and 0.05% FA. SCX buffer A was made of 5 mM KH_2_PO_4_ (Merck, Germany), 30% ACN and 0.05% FA, pH 2.7; SCX buffer B consisted of 350 mM KCl (Merck, Germany), 5 mM KH_2_PO_4_, 30% ACN and 0.05% FA, pH 2.7. The gradient was performed as follows: 0% B for 10 min, 0–85% B in 35 min, 85–100% B in 6 min and 100% B for 4 min. After injection of 200 μg of labeled lysate, a total of 45 fractions were collected, dried in a vacuum centrifuge and stored at −80°C.

### Phosphoproteome analysis

The labeled peptides were reconstituted in 10% formic acid prior to fractionation using SCX as described previously (Hennrich *et al*, 2013). The experiments were performed on an Agilent 1100/1200 HPLC system (Agilent Technologies, Germany). Peptides corresponding to 3 mg of tryptic digested lysate were loaded onto a C_18_ trap column (strata-x 33 μm Polymeric Reversed phase, 50 × 4.6 mm, Phenomenex, The Netherlands) for 5 min at 300 μl/min using aqueous 0.05% FA as solvent. Subsequently, peptides were eluted for 5 min from the trapping column with 80% ACN containing 0.05% FA onto a polysulfoethyl A column 200 × 2.1 mm, 5 μm particles and 200 Å pore size (PolyLC Inc., Columbia, MD) at the same flow rate. Separation was performed using a nonlinear 65 min gradient: isocratic for 2 min at 100% solvent A (5 mM KH_2_PO_4_, 30% ACN and 0.05% FA, pH 2.7); from 2 to 10 min at 3% solvent B (5 mM KH_2_PO_4_, 30% ACN, 350 mM KCl and 0.05% FA at pH 2.7); from 10 to 40 min a gradient to 35% solvent B; and from 40 to 45 min to 100% solvent B. The column was subsequently washed for 10 min with solvent B and finally equilibrated with 100% solvent A. Fractions were collected in 1-min intervals for the first 40 min and for 3-min intervals in the last 15 min. The fractions were desalted using Sep-Pak Vac C_18_ cartridge (3 cc/200 mg, Waters), and the eluted peptides were dried down and stored at −80°C for phosphopeptide enrichment by Ti^4+^-IMAC.

### Phosphopeptide enrichment

Ti^4+^-IMAC material was prepared and used essentially as previously described (Zhou *et al*, 2013). The prepared Ti^4+^-IMAC beads were loaded onto GELoader tips (Eppendorf) using a C_8_ plug to approximately 1–2 cm length of material. The enrichment procedure for all SCX fractions was as follows: The Ti^4+^-IMAC material was pre-equilibrated two times with 50 μl of Ti^4+^-IMAC loading buffer (80% ACN, 6% trifluoroacetic acid (TFA)). Next, each SCX fraction was resuspended in 50 μl of loading buffer and loaded onto the equilibrated GELoader tips. Then, the Ti^4+^-IMAC material was washed with 50 μl wash buffer A (50% ACN, 0.5% TFA, 200 mM NaCl) and subsequently with 50 μl wash buffer B (50% ACN, 0.1% TFA). Bound peptides were first eluted by 30 μl of 10% ammonia into 30 μl of 10% FA. Finally, the remainder of the peptides was eluted with 2 μl of 80% ACN, 2% FA. The collected eluate was further acidified by adding 3 μl of 100% FA and subsequently stored at −80°C for LC-MS/MS analysis.

### LC/MS/MS analysis

The phosphopeptides and the 3^+^ SCX fractions of the whole proteome analysis were analyzed on LTQ-Orbitrap Velos or Elite Mass Spectrometer equipped with an electron transfer dissociation (ETD) source (Thermo Fisher, Germany) and connected to an Easy UHPLC system (both Thermo Fisher Scientific, Germany). The columns were made in-house from either Aqua™ C_18_ (5 μm, Phenomenex, Torrance, USA; 20 mm × 100 μm i.d.) for the trap column or Zorbax C_18_ (1.8 μm, Agilent; 38 cm × 50 μm i.d.) for the analytical column (Cristobal *et al*, 2012). Mobile-phase buffers for nLC separation consisted of 0.1% formic acid in water (solvent A) and 100% ACN/0.1% formic acid (solvent B). The peptides were eluted during a 3-h gradient and directly sprayed into the mass spectrometer. The flow rate was set at 100 nl/min, and the LC gradient was as follows: 7–30% solvent B within 151 min, 30–100% solvent B within 3 min, 100% solvent B for 2 min and 7% solvent B for 23 min. Nano spray was achieved with an in-house pulled and gold-coated fused silica capillary (360 mm o.d.; 20 mm i.d.; 10 mm tip i.d.) and an applied voltage of 1.7 kV. The mass spectrometer was programmed in a data-dependent decision tree acquisition mode and was configured to perform a Fourier transform survey scan from 350 to 1,500 *m/z* (resolution 60,000) followed by higher collision energy dissociation (HCD; 32% normalized collision energy, resolution 15,000) or ETD fragmentation of the 20 most intense peaks depending on the charge state and *m*/*z* of the precursor as previously described (Frese *et al*, 2011). Supplemental activation was enabled for ETD. The 2^+^ SCX fractions of the whole proteome analysis were analyzed on a Q-Exactive mass spectrometer connected to an Easy UHPLC system (both Thermo Fisher Scientific, Germany). The mass spectrometer was programmed in the data-dependent acquisition mode and was configured to perform a Fourier transform survey scan from 350 to 1,500 *m/z* (resolution 35,000) followed by higher collision energy dissociation fragmentation of the 20 most intense peaks (25% normalized collision energy at a target value of 50,000 ions, resolution 17,500).

### Data processing

Raw data were analyzed by MaxQuant (version 1.3.0.5) (Cox & Mann, 2008). Andromeda (Cox *et al*, 2011) was used to search the MS/MS data against the human UniProt database (20,247 entries, released 2012_02) complemented with a list of common contaminants and concatenated with the reversed version of all sequences. Trypsin/P was chosen as cleavage specificity allowing two missed cleavages. Carbamidomethylation (C) was set as a fixed modification, while oxidation (M) and phosphorylation of STY were used as variable modifications. For dimethyl labeling, DimethylLys0 and DimethylNter0 were set as light labels, DimethylLys4 and DimethylNter4 were set as medium labels, and DimethylLys8 and DimethylNter8 were set as heavy labels. Peptide identification was based on a search with a mass deviation of the precursor ion of up to 6 ppm, and the allowed fragment mass deviation was set to 0.05 Da for FTMS and 0.6 Da for ITMS. Data filtering was carried out using the following parameters: Peptide and protein FDRs were set to 1%, minimum peptide length was set to 6, and Andromeda minimum score was set to 60 [≅ Mascot score 20 (Cox *et al*, 2011)]. The reverse and common contaminant hits were removed from MaxQuant output. Protein quantification was performed by using only unmodified peptides and oxidation (M); the re-quantify option was enabled. Only unique peptides with at least two ratio counts were used for protein quantification.

### Data availability

The mass spectrometry proteomics data have been deposited to the ProteomeXchange Consortium (http://proteomecentral.proteomexchange.org) via the PRIDE partner repository (Vizcaino *et al*, 2013) with the dataset identifier PXD000497. PX reviewer account: username: review83857; password: 4G97r7 g3.

### Statistics

To filter for those proteins that show (or have) a consistent abundance level over three independent biological replicates (1 day/control and 3 days/control), we applied a one-sample *t*-test against 0 (no abundance change). Only those proteins that had a *P*-value* *< 0.05 were considered. A two-sample *t*-test was performed to assess protein ratio differences between the two groups (3 days/control versus 1 day/control) and used as a filter to extract those proteins or phosphopeptides that show significant abundance differences (*P*-value* *< 0.05). In addition to the statistical filters, only proteins and phosphopeptides with an arbitrary cutoff ratio ≥ 1.5 or ≤ −1.5 fold changes were considered. Furthermore, phosphopeptides were required to have a phosphosite location probability ≥ 0.75.

### Reactome analysis

The significant entries at protein level (with a fold change ≥ 1.5 or ≤ −1.5) were analyzed by Cytoscape 2.8 (Smoot *et al*, 2011) using Reactome (Haw *et al*, 2011) and Cerebral as plugins (Barsky *et al*, 2007). Predicted interactions were removed from the analysis. Protein location was retrieved from UniProt database, and in case a protein had multiple locations, one was arbitrarily chosen to insert in Cerebral (Supplementary Table S4).

### Gene ontology enrichment analysis by BiNGO

BiNGO Cytoscape plugin was used to perform gene ontology (GO) slim enrichment analysis (Maere *et al*, 2005). For the proteome analysis, the software was run using both target (significant regulated proteins with a fold change ≥ 1.5 or ≤ −1.5) and background list (the complete list of identified proteins and phosphoproteins), to calculate enrichment of biological processes across the target list. The same procedure was performed for the phosphoproteome. A hypergeometric test was performed to test for enrichment, and Benjamini & Hochberg false discovery rate correction was applied to correct for multiple testing. Enriched biological processes below the corrected *P*-value threshold of 0.05 were considered.

## Supporting Information

Supplementary Table Legends

Review Process File
