# Supplementary material for: ROCK1 is a potential combinatorial drug target for BRAF mutant melanoma
Source: Mol Syst Biol. 2014 Dec 23;10(12):772. doi: 10.15252/msb.20145450 (PMC4300494; doi:10.15252/msb.20145450)
Supplement: Supplementary file 1 [file msb0010-0772-sd1.pdf]

Figure S1

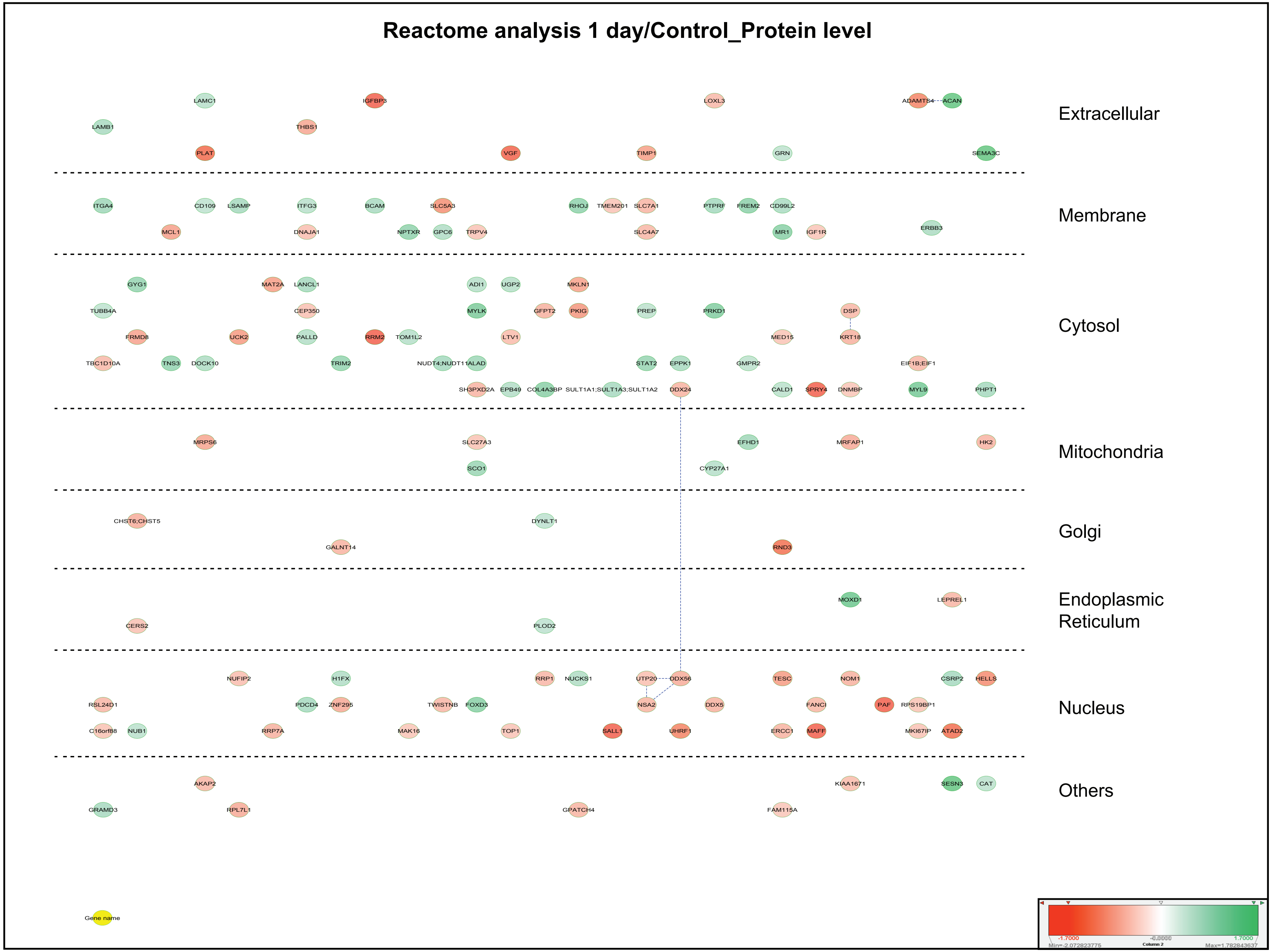
Reactome analysis 1 day/Control_Protein level
Extracellular
LAMC1
IGFBP3
LOXL3
ADAMTS4
ACAN
LAMB1
THBS1
PLAT
VGF
TIMP1
GRN
SEMA3C
Membrane
ITGA4
CD109
LSAMP
ITFG3
BCAM
SLC5A3
RHOJ
TMEM201
SLC7A1
PTPRF
FREM2
CD99L2
MCL1
DNAJA1
NPTXR
GPC6
TRPV4
SLC4A7
MR1
IGF1R
ERBB3
Cytosol
GYG1
MAT2A
LANCL1
ADI1
UGP2
MKLN1
TUBB4A
CEP350
MYLK
GFPT2
PKIG
PREP
PRKD1
DSP
FRMD8
UCK2
PALLD
RRM2
TOM1L2
LTV1
MED15
KRT18
TBC1D10A
TNS3
DOCK10
TRIM2
NUDT4;NUDT11
ALAD
STAT2
EPPK1
GMPR2
EIF1B;EIF1
SH3PXD2A
EPB49
COL4A3BP
SULT1A1;SULT1A3;SULT1A2
DDX24
CALD1
SPRY4
DNMBP
MYL9
PHPT1
Mitochondria
MRPS6
SLC27A3
EFHD1
MRFAP1
HK2
SCO1
CYP27A1
Golgi
CHST6;CHST5
DYNLT1
GALNT14
RND3
Endoplasmic Reticulum
MOXD1
LEPREL1
CERS2
PLOD2
Nucleus
NUFIP2
H1FX
RRP1
NUCKS1
UTP20
DDX56
TESC
NOM1
CSRP2
HELLS
RSL24D1
PDCD4
ZNF295
TWISTNB
FOXD3
NSA2
DDX5
FANCI
PAF
RPS19BP1
C16orf88
NUB1
RRP7A
MAK16
TOP1
SALL1
UHRF1
ERCC1
MAFF
MKI67IP
ATAD2
Others
AKAP2
KIAA1671
SESN3
CAT
GRAMD3
RPL7L1
GPATCH4
FAM115A
Gene name
-1.7000
-0.0000
1.7000
Min=-2.072823775
Column 2
Max=1.782843637

Reactome analysis 3 days/Control_Protein level

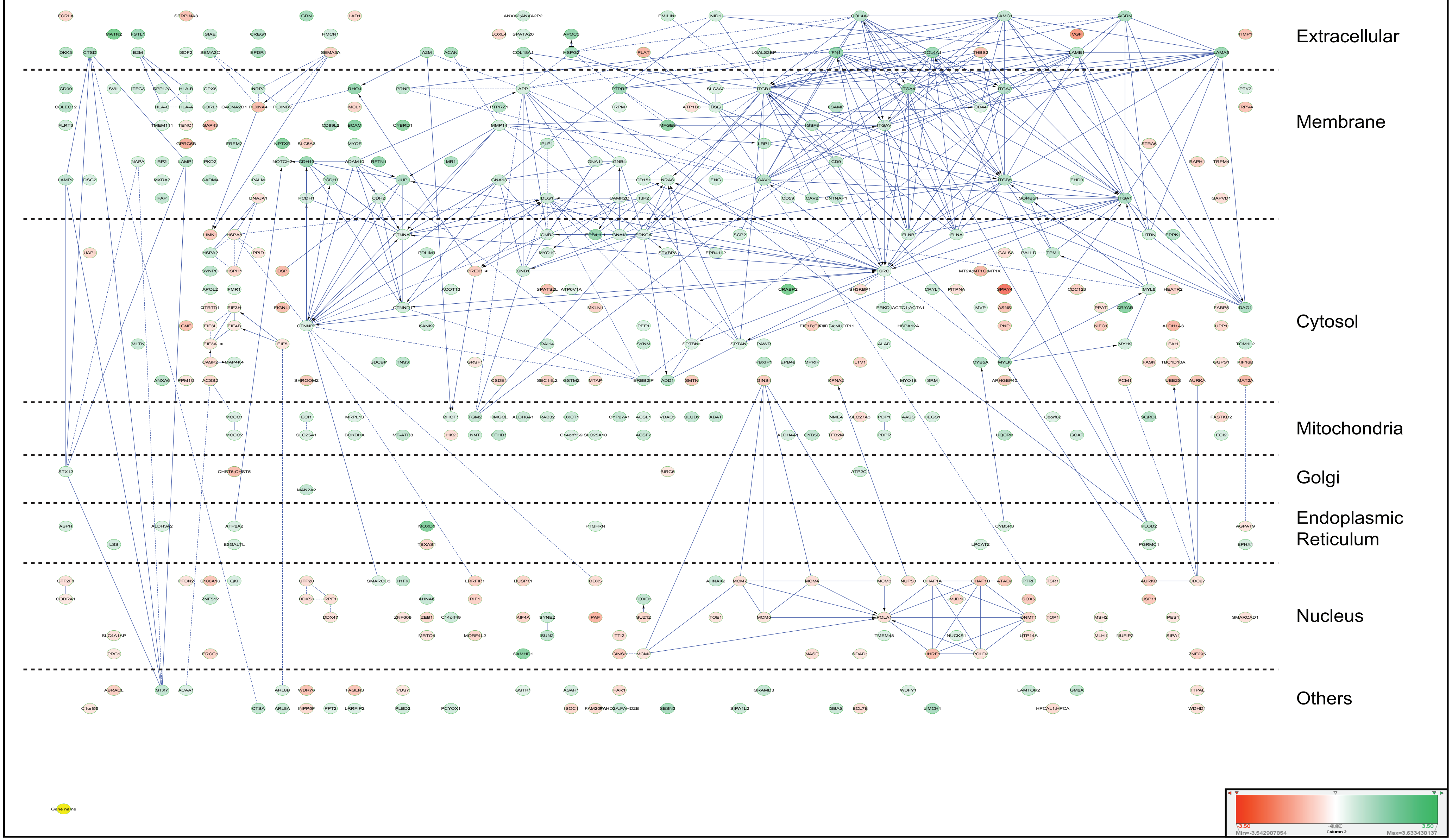

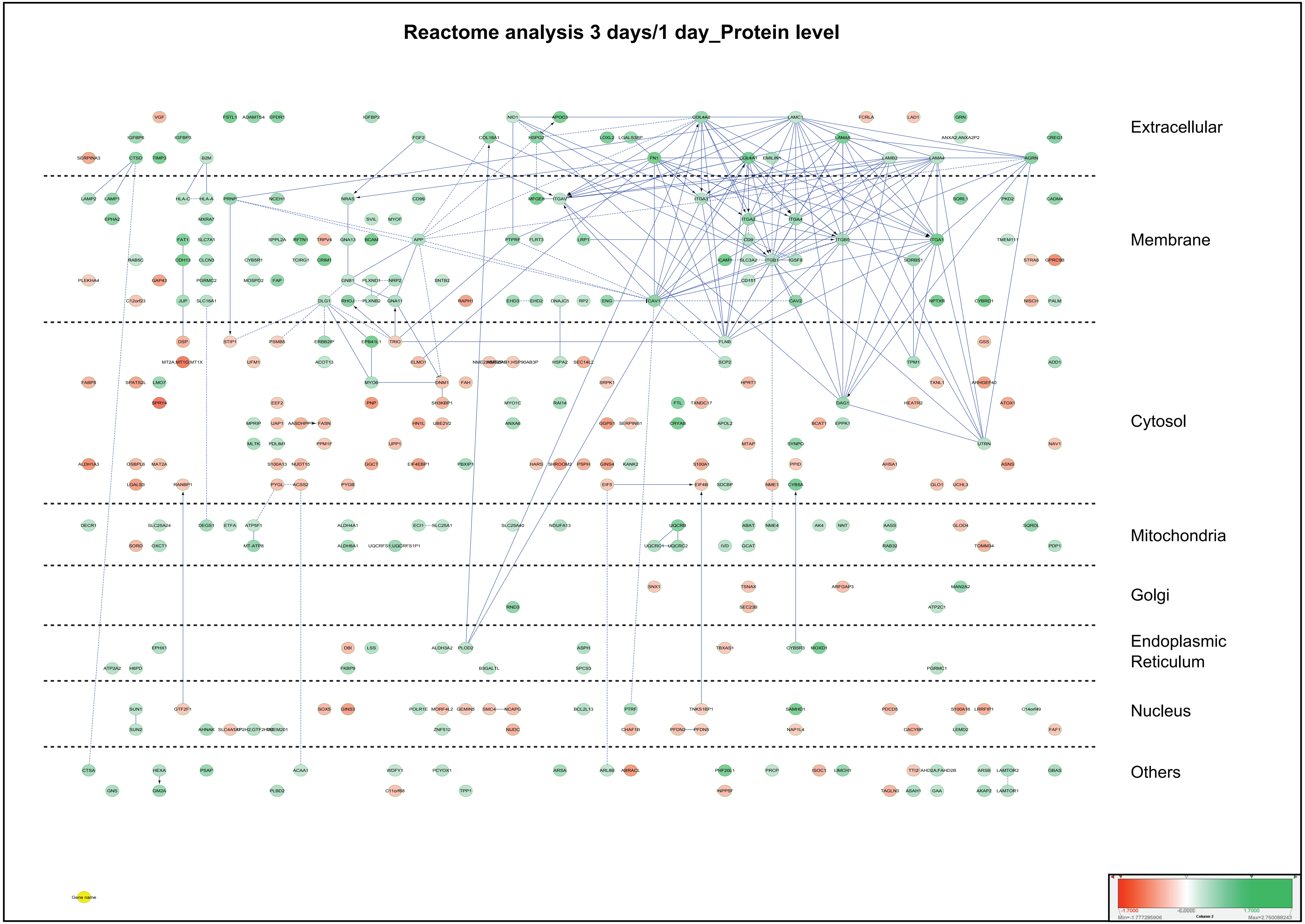

Reactome analysis 3 days/1 day_Protein level
Extracellular
Membrane
Cytosol
Mitochondria
Golgi
Endoplasmic Reticulum
Nucleus
Others
VGF
FSTL1
ADAMTS4
EPDR1
IGFBP2
NID1
APOC3
COL4A2
LAMC1
FCRLA
LAD1
GRN
IGFBP6
IGFBP3
FGF2
COL18A1
HSPG2
LOXL2
LGALS3BP
LAMA5
ANXA2;ANXA2P2
CREG1
SERPINA3
CTSD
TIMP3
B2M
FN1
COL4A1
EMILIN1
LAMB2
LAMA4
AGRN
LAMP2
LAMP1
HLA-C
HLA-A
PRNP
NCEH1
NRAS
CD99
MFGE8
ITGAV
ITGA3
SORL1
PKD2
CADM4
EPHA2
MXRA7
SVIL
MYOF
ITGA2
ITGA4
FAT1
SLC7A1
SPPL2A
RFTN1
TRPV4
GNA13
BCAM
APP
PTPRF
FLRT3
LRP1
CD9
ITGB5
ITGA1
TMEM111
RAB5C
CDH13
CLCN3
CYB5R1
TCIRG1
CRIM1
ICAM1
SLC3A2
ITGB1
IGSF8
SORBS1
STRA6
GPRC5B
PLEKHA4
GAP43
PGRMC2
MOSPD2
FAP
GNB1
PLXND1
NRP2
SNTB2
CD151
C12orf23
JUP
SLC16A1
DLG1
RHOJ
PLXNB2
GNA11
RAPH1
EHD3
EHD2
DNAJC5
RP2
ENG
CAV1
CAV2
NPTXR
CYBRD1
NISCH
PALM
DSP
STIP1
PSMB5
ERBB2IP
EPB41L1
TRIO
FLNB
GSS
MT2A;MT1G;MT1X
UFM1
ACOT13
ELMO1
HSPA2
SEC14L2
SCP2
TPM1
ADD1
FABP5
SPATS2L
LMO7
MYO6
DNM1
FAH
SRPK1
HPRT1
TXNL1
ARHGEF40
SPRY4
EEF2
PNP
SH3KBP1
MYO1C
RAI14
FTL
TXNDC17
DAG1
HEATR2
ATOX1
MPRIP
UAP1
AASDHPPT
FASN
HN1L
UBE2V2
ANXA6
GGPS1
SERPINB1
CRYAB
APOL2
BCAT1
EPPK1
MLTK
PDLIM1
PPM1F
UPP1
MTAP
SYNPO
UTRN
NAV1
ALDH1A3
OSBPL6
MAT2A
S100A13
NUDT15
GGCT
EIF4EBP1
PBXIP1
HARS
SHROOM2
PSPH
GINS4
KANK2
S100A1
PPID
AHSA1
ASNS
LGALS3
RANBP1
PYGL
ACSS2
PYGB
EIF5
EIF4B
SDCBP
NME1
CYB5A
GLO1
UCHL3
DECR1
SLC25A24
DEGS1
ETFA
ATP5F1
ALDH4A1
ECI1
SLC25A1
SLC25A40
NDUFA13
UQCRB
ABAT
NME4
AK4
NNT
AASS
GLOD4
SQRDL
SORD
OXCT1
MT-ATP8
ALDH6A1
UQCRFS1;UQCRFS1P1
UQCRC1
UQCRC2
IVD
GCAT
RAB32
TOMM34
PDP1
SNX1
TSNAX
ARFGAP3
MAN2A2
RND3
SEC23B
ATP2C1
EPHX1
DBI
LSS
ALDH3A2
PLOD2
ASPH
TBXAS1
CYB5R3
MOXD1
ATP2A2
H6PD
FKBP9
B3GALTL
SPCS3
PGRMC1
SUN1
GTF2F1
SOX5
GINS3
POLR1E
MORF4L2
GEMIN5
SMC4
NCAPG
BCL2L13
PTRF
TNKS1BP1
SAMHD1
PDCD5
S100A16
LRRFIP1
C14orf49
SUN2
AHNAK
CHAF1B
PFDN2
PFDN5
NAP1L4
CACYBP
LEMD2
FAF1
ZNF512
NUDC
CTSA
HEXA
PSAP
ACAA1
WDFY1
PCYOX1
ARSA
ARL8B
ABRACL
PHF20L1
PRCP
ISOC1
LIMCH1
ARSB
LAMTOR2
GBAS
GNS
GM2A
PLBD2
C11orf68
TPP1
INPP5F
TAGLN3
ASAH1
GAA
AKAP2
LAMTOR1
Gene name
-1.7000
-0.0000
1.7000
Min=-1.777285906
Column 2
Max=2.750088243

Figure S1. Reactome analysis at p rotein level at 1d/control; 3d/control; and 3d/1d. The $\log_2$ protein ratio is represented by the color scale where the up-regulated proteins are highlighted in green, while the down-regulated are labeled in red.
