# Supplementary material for: ROCK1 is a potential combinatorial drug target for BRAF mutant melanoma
Source: Mol Syst Biol. 2014 Dec 23;10(12):772. doi: 10.15252/msb.20145450 (PMC4300494; doi:10.15252/msb.20145450)
Supplement: Supplementary file 2 [file msb0010-0772-sd2.pdf]

Figure S2

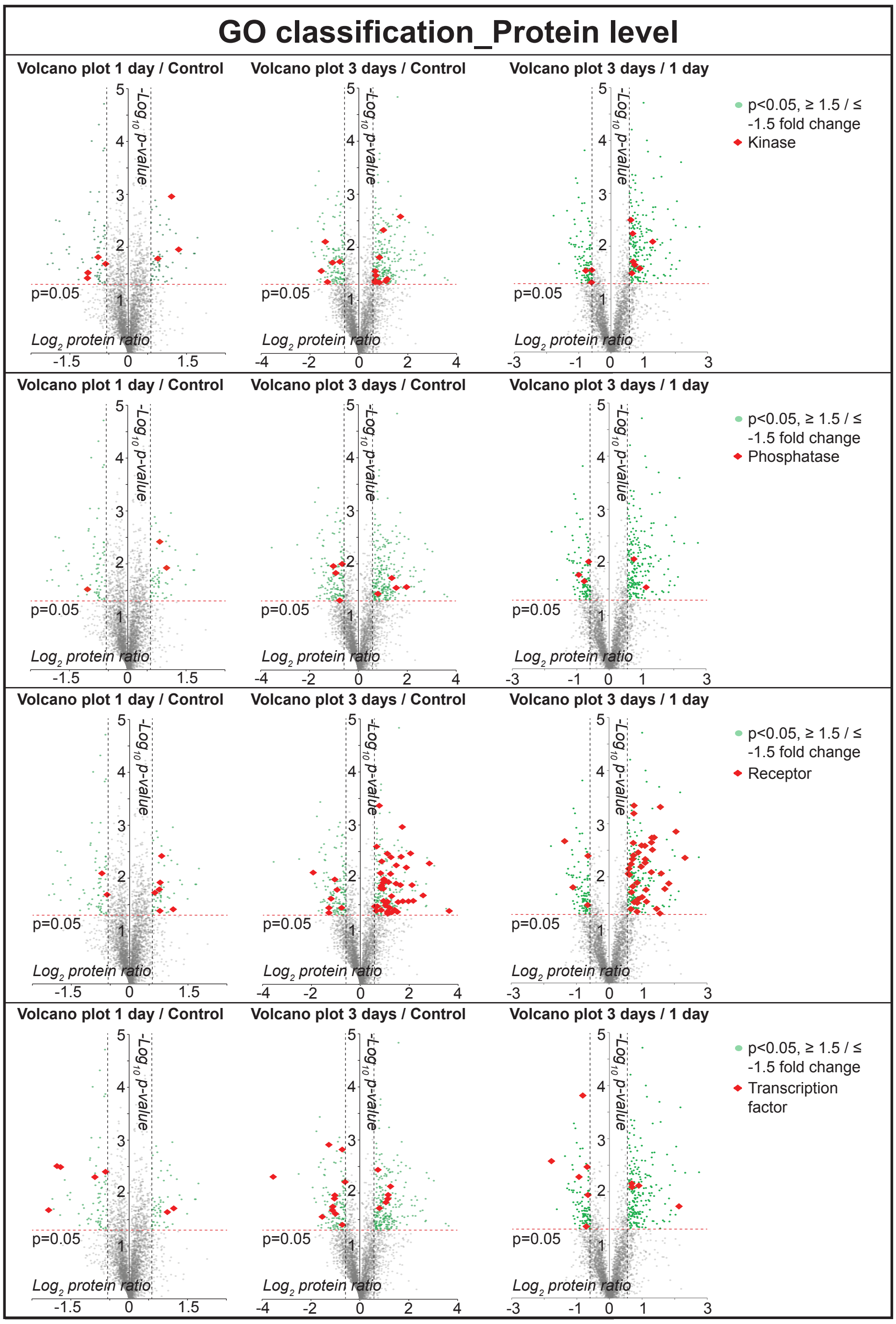

Figure S2. GO classification using Panther. Proteins belonging to the four categories (kinase; phosphatase; receptor; transcription factor) that are over-represented among the significant entries are labeled by a red diamond in each volcano plot at each time point.
