# Supplementary material for: ROCK1 is a potential combinatorial drug target for BRAF mutant melanoma
Source: Mol Syst Biol. 2014 Dec 23;10(12):772. doi: 10.15252/msb.20145450 (PMC4300494; doi:10.15252/msb.20145450)
Supplement: Supplementary file 3 [file msb0010-0772-sd3.pdf]

Figure S3

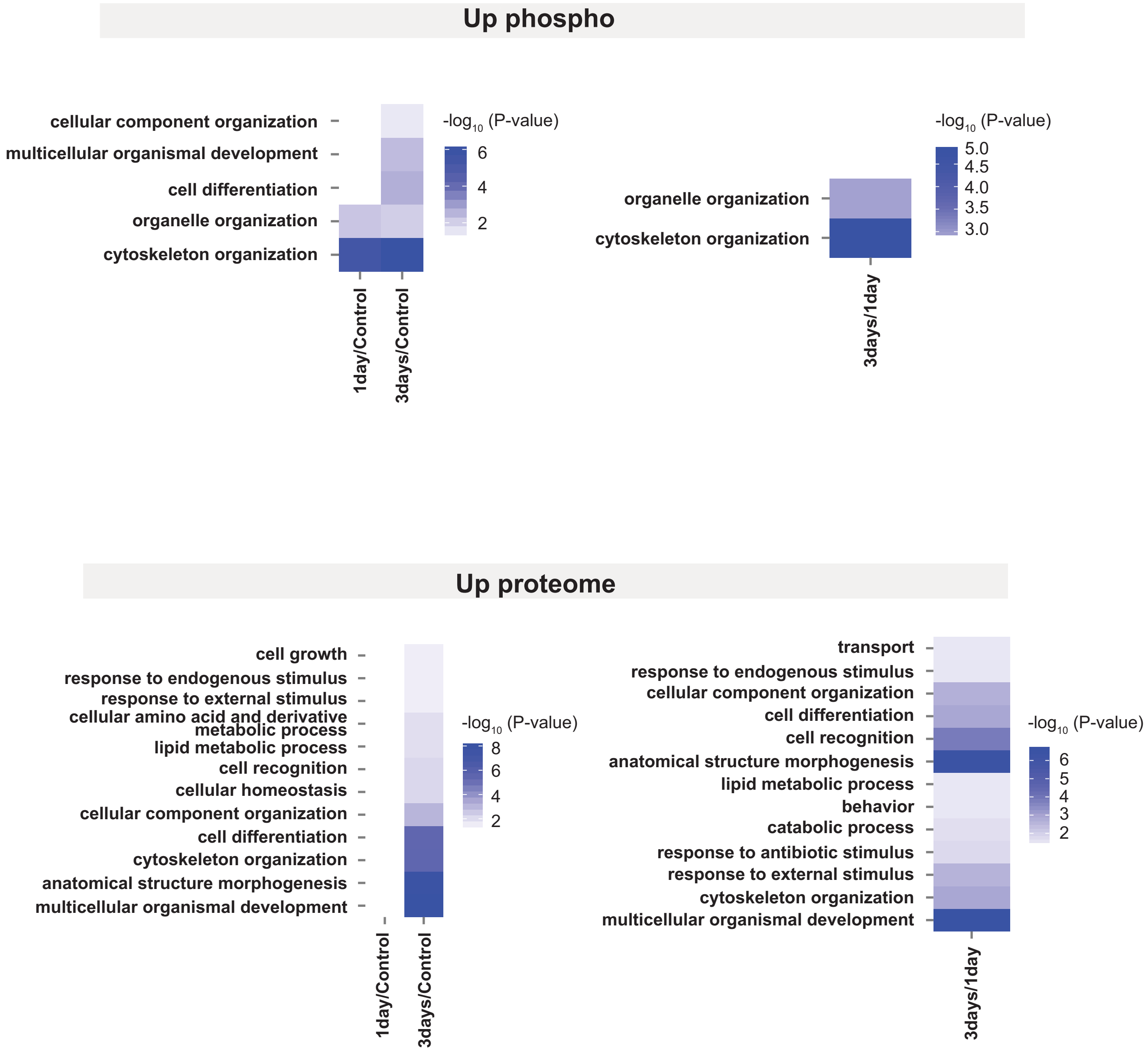
Up phospho
cellular component organization
multicellular organismal development
cell differentiation
organelle organization
cytoskeleton organization
1day/Control
3days/Control
-log10 (P-value)
6
4
2
organelle organization
cytoskeleton organization
3days/1day
-log10 (P-value)
5.0
4.5
4.0
3.5
3.0
Up proteome
cell growth
response to endogenous stimulus
response to external stimulus
cellular amino acid and derivative metabolic process
lipid metabolic process
cell recognition
cellular homeostasis
cellular component organization
cell differentiation
cytoskeleton organization
anatomical structure morphogenesis
multicellular organismal development
1day/Control
3days/Control
-log10 (P-value)
8
6
4
2
transport
response to endogenous stimulus
cellular component organization
cell differentiation
cell recognition
anatomical structure morphogenesis
lipid metabolic process
behavior
catabolic process
response to antibiotic stimulus
response to external stimulus
cytoskeleton organization
multicellular organismal development
3days/1day
-log10 (P-value)
6
5
4
3
2

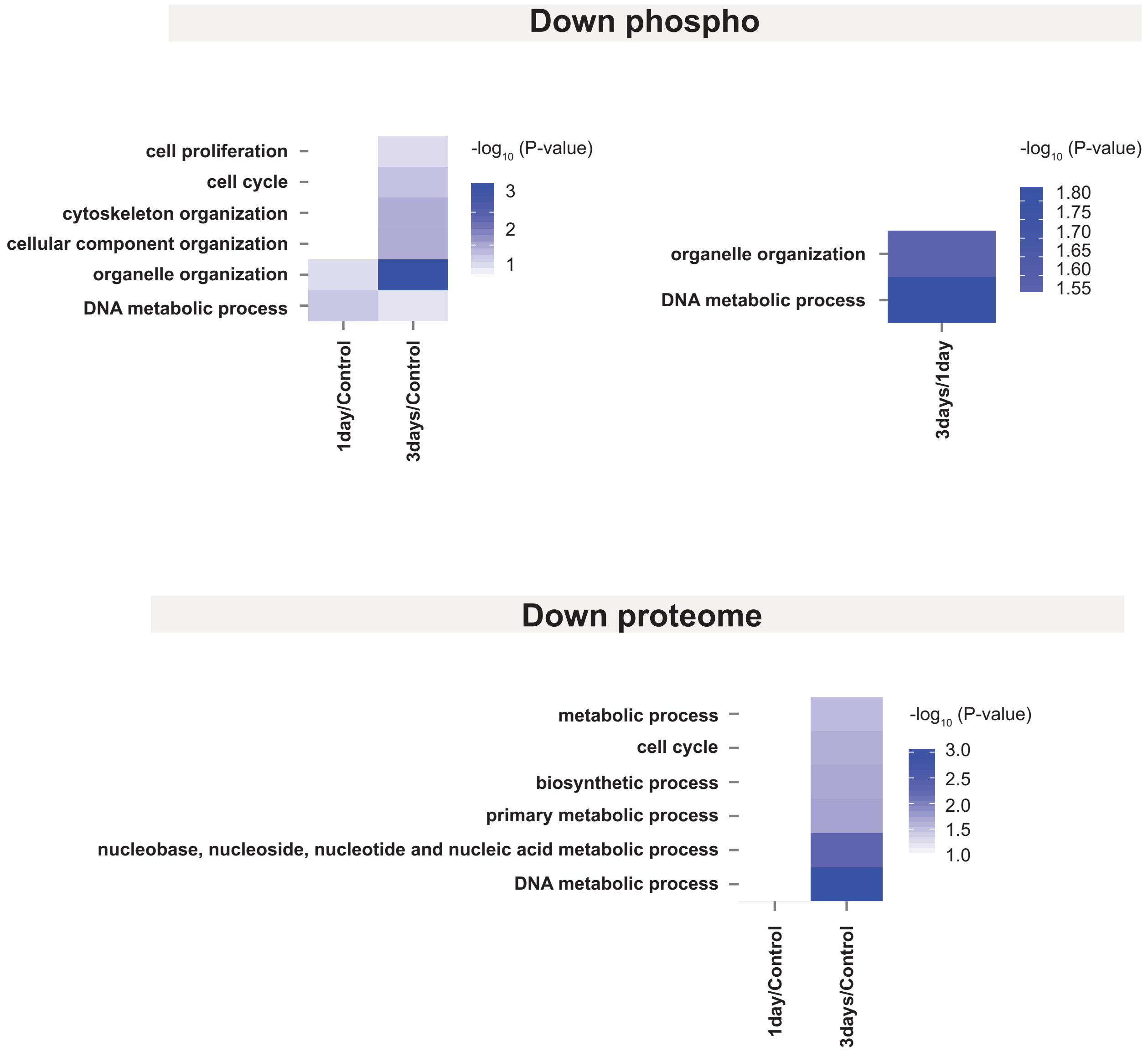

Figure S3. GO slim enrichment analysis. GO slim enrichment analysis using BiNGO as cytoscape plugin.
