# Supplementary material for: ROCK1 is a potential combinatorial drug target for BRAF mutant melanoma
Source: Mol Syst Biol. 2014 Dec 23;10(12):772. doi: 10.15252/msb.20145450 (PMC4300494; doi:10.15252/msb.20145450)
Supplement: Supplementary file 4 [file msb0010-0772-sd4.pdf]

Figure S4

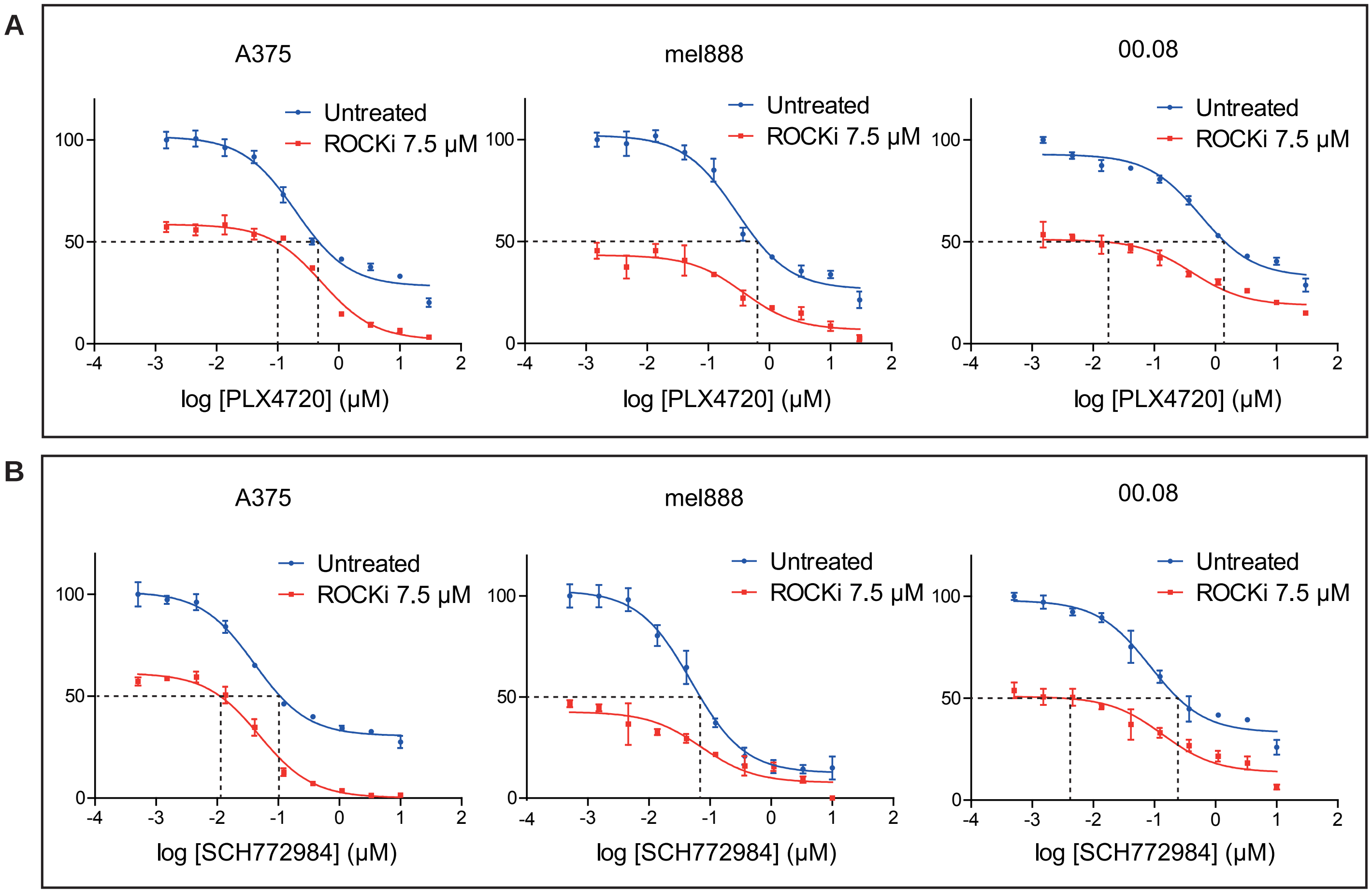

Figure S4. Targeted ROCK inhibition increases the toxicity of inhibitors of the MAPK pathway. A) Cells were treated with dilution series of PLX4720 either alone or in combination with the ROCK inhibitor GSK269962A. After three days cell viability was determined with a cell titer blue assay and represented on the y-axis. Error bars represent standard error of the mean of one representative experiment performed in triplicate. Dashed lines indicate change in IC50. B) Cells were treated as described in A, but treated with a dilution series of the ERK inhibitor SCH772984. Error bars represent standard error of the mean of one representative experiment performed in triplicate. Dashed lines indicate chan-ges in IC50.
