# Supplementary material for: ROCK1 is a potential combinatorial drug target for BRAF mutant melanoma
Source: Mol Syst Biol. 2014 Dec 23;10(12):772. doi: 10.15252/msb.20145450 (PMC4300494; doi:10.15252/msb.20145450)
Supplement: Supplementary file 9 [file msb0010-0772-sd9.docx]

**Legends to Supplementary Tables**

**Table S1.** Statistical significant proteins/phosphosites (p<0.05) with a fold change ≥ 1.5 or ≤ -1.5. WP = whole proteome.

**Table S2.** GO analysis using Panther.

**Table S3.** GO slim enrichment analysis.

**Table S4.** shRNAs identified in the BRAFi and ERKi screens. Genes identified with multiple hairpins are highlighted in red (ERKi screen), pink (BRAFi screen) or purple (overlap of both screens).
