# Supplementary material for: ROCK1 is a potential combinatorial drug target for BRAF mutant melanoma
Source: Mol Syst Biol. 2014 Dec 23;10(12):772. doi: 10.15252/msb.20145450 (PMC4300494; doi:10.15252/msb.20145450)
Supplement: Supplementary file 10 [file msb0010-0772-sd10.pdf]

molecular systems biology

ROCK1 is a potential combinatorial drug target for BRAF mutant melanoma

Marjon A. Smit, Gianluca Maddalo, Kylie Greig, Linsey M. Raaijmakers, Patricia A. Possik, Bas van Breukelen, Salvatore Cappadona, Albert J.R. Heck, A.F. Maarten Altelaar, Daniel S. Peeper

Corresponding author: Daniel S. Peeper, Netherlands Cancer Institute

Review timeline:

Submission date:	26 May 2014
Editorial Decision:	17 July 2014
Revision received:	12 October 2014
Editorial Decision:	20 November 2014
Accepted:	04 December 2014

Editor: Maria Polychronidou

Transaction Report:

(Note: With the exception of the correction of typographical or spelling errors that could be a source of ambiguity, letters and reports are not edited. The original formatting of letters and referee reports may not be reflected in this compilation.)

1st Editorial Decision 17 July 2014

Thank you again for submitting your work to Molecular Systems Biology. I would like to apologize for the delay in getting back to you, which was due to the late arrival of one of the reports. We have now heard back from the referees who agreed to evaluate your manuscript. As you will see below, the referees acknowledge that the presented findings are potentially interesting. However, they raise a series of concerns, which should be carefully addressed in a revision of the manuscript. The recommendations provided by the referees are very clear in this regard.

While the <I>in vivo</I> validation suggested by reviewer #2 is not mandatory, we certainly welcome inclusion of such data, as they would significantly strengthen the impact of the study.

On a more editorial level, we would like to draw your attention to the following:
- We would like to ask you to provide the results of the genetic screens in a Supplementary Table.
- Please include the links and dataset identifiers for the proteomics data in the "Data Availability" section of your manuscript.

If you feel you can satisfactorily deal with these points and those listed by the referees, you may wish to submit a revised version of your manuscript. Please attach a covering letter giving details of the way in which you have handled each of the points raised by the referees. A revised manuscript will be once again subject to review and you probably understand that we can give you no guarantee at this stage that the eventual outcome will be favorable.

© European Molecular Biology Organization

Reviewer #1:

The authors describe a study that integrates the results of proteomic, phospho-proteomic and kinome shRNA screening following treatment of 04.01 melanoma cancer cells with the BRAF inhibitor PLX4720 in order to identify drug targets that sensitize melanoma cells to BRAF inhibition. They also performed a second shRNA screen in the presence of the ERK inhibitor SCH772984 since many resistance mechanisms to BRAF inhibition involve re-activation of ERK.

The authors found many proteins to be induced upon PLX4720 treatment that are known to be involved in BRAF inhibitor resistance including FOXD3 and ERBB3. Integration of proteomic and functional genomic analysis revealed that regulation of ROCK signalling was prevalent upon PLX4720 treatment. For example, RND3 is a negative regulator of ROCK1 and was significantly down-regulated. Additionally, a number of integrins were up-regulated including ITGB1, which has been shown to activate the RhoA-ROCK pathway in colon carcinoma cells. The authors go on to validate that ROCK1 silencing through genetic and chemical inhibition sensitizes melanoma cells to BRAF and ERK inhibition.

Overall, the manuscript is clearly written and all the experiments are well controlled. The results have significant potential impact. The synthetic lethal interaction that the authors describe between ROCK1 and BRAF/ERK is exciting and for this reason I would encourage the authors to demonstrate this synthetic lethal effect in a handful of additional melanoma cell lines established from different patient tumors, and that are sensitive to BRAF inhibitors. This obviously raises the question about additional tumor types and whether this genetic interaction is consistent across all tumor types that have oncogenic BRAF mutations?

Reviewer #2:

elegant and appropriate phosphoproteomic and functional genomic platforms to identify drug targets whose co-inhibition sensitizes BRAF mutant melanoma cells to BRAF inhibition. Mass spec data revealed some proteins to be induced with the BRAF inhibitor PLX4720 treatment, some of them known to be involved in BRAF inhibitor resistance (FOXD3 and ErbB3). Some other proteins were downregulated such as Rnd3, a negative regulator of ROCK1 kinase. This mass spec data integrated with the genomic data, obtained by shRNA screens using a kinome library, revealed that ROCK1 is a potential drug target for BRAF mutant melanoma. Targeting ROCK1 led to increased melanoma cell elimination when combined with BRAF or ERK inhibitor treatment. Moreover, a ROCK inhibitor showed augmented melanoma cell death upon BRAF or ERK inhibition. Authors claimed that ROCK1 should be explored as a target in combination with current BRAF mutant melanoma therapies.

This is an interesting study that potentially uncovers a new target for BRAF mutant melanoma treatment. However, some points need to be addressed to improve the manuscript and to support the conclusions.

Major:

1. Phosphoproteomic studies are very variable in terms of phosphopeptides recovery after SCX processing and therefore technical replicates are crucial to support the relevance of the findings. Although authors based their results in 3 biological replicates, it would strengthen the study if the key phospho-proteome changes were validated by Western blot.
2. MS/MS data analysis against the human Uniprot database renders a probabilistic identification of the proteins. Therefore, authors should also validate more total proteins to support the findings. The only protein validated in the manuscript is ROCK1.
3. The clinical importance of this finding is not clear. The study would be strengthened if the findings were validated by IHC stain of ROCK1 in melanoma samples with BRAF mutation.
4. The study would be strengthened by in vivo validation using a combination of ROCK1 and BRAF inhibitors to show that their findings hold true in a clinically relevant setting.

Minor points:
ï Figure 4B-C and Figure5 A-B, it would be better to move the Y axis to the intersection with (-3) in

© European Molecular Biology Organization

the X axis. It would also be helpful to show in the figure the change in the IC50 value achieved with each treatment.

1st Revision - authors' response 12 October 2014

Reviewer #1

We would like to thank the reviewer for his/her valuable comments, which we have addressed as outlined below:

1. *The authors describe a study that integrates the results of proteomic, phospho-proteomic and kinome shRNA screening following treatment of 04.01 melanoma cancer cells with the BRAF inhibitor PLX4720 in order to identify drug targets that sensitize melanoma cells to BRAF inhibition. They also performed a second shRNA screen in the presence of the ERK inhibitor SCH772984 since many resistance mechanisms to BRAF inhibition involve re-activation of ERK.*

   The authors found many proteins to be induced upon PLX4720 treatment that are known to be involved in BRAF inhibitor resistance including FOXD3 and ERBB3. Integration of proteomic and functional genomic analysis revealed that regulation of ROCK signalling was prevalent upon PLX4720 treatment. For example, RND3 is a negative regulator of ROCK1 and was significantly down-regulated. Additionally, a number of integrins were up-regulated including ITGB1, which has been shown to activate the RhoA-ROCK pathway in colon carcinoma cells. The authors go on to validate that ROCK1 silencing through genetic and chemical inhibition sensitizes melanoma cells to BRAF and ERK inhibition.

   Overall, the manuscript is clearly written and all the experiments are well controlled. The results have significant potential impact. The synthetic lethal interaction that the authors describe between ROCK1 and BRAF/ERK is exciting and for this reason I would encourage the authors to demonstrate this synthetic lethal effect in a handful of additional melanoma cell lines established from different patient tumors, and that are sensitive to BRAF inhibitors.

We have now examined three additional cell lines derived from melanoma patient samples with a $BRAF^{E600}$ mutation, bringing the total number of tested cell lines to six. The new data are shown in Figure E4 and strengthen our conclusions about a synthetic lethal interaction between ROCK1 and BRAF/ERK.

2. *This obviously raises the question about additional tumor types and whether this genetic interaction is consistent across all tumor types that have oncogenic BRAF mutations?*

This is indeed an interesting question, however not all tumor types that harbor a BRAF mutation are sensitive to BRAF targeted therapy. For example, BRAF inhibitors are barely effective in colon cancer (Kopetz, S *et al.*, J. Clin. Oncol. 28 abstract 3534, 2010). As the complexities of BRAF signaling are still being mapped out in other tumor types, we have focused our current study on melanoma cells. We hope that the referee will agree that albeit of interest, this topic is beyond the scope of this study.

Reviewer #2

We would like to thank the reviewer for his/her valuable comments, which we have addressed as outlined below:

Elegant and appropriate phosphoproteomic and functional genomic platforms to identify drug targets whose co-inhibition sensitizes BRAF mutant melanoma cells to BRAF inhibition. Mass spec data revealed some proteins to be induced with the BRAF inhibitor PLX4720 treatment, some of

© European Molecular Biology Organization

them known to be involved in BRAF inhibitor resistance (FOXD3 and ErbB3). Some other proteins were downregulated such as Rnd3, a negative regulator of ROCK1 kinase. This mass spec data integrated with the genomic data, obtained by shRNA screens using a kinome library, revealed that ROCK1 is a potential drug target for BRAF mutant melanoma. Targeting ROCK1 led to increased melanoma cell elimination when combined with BRAF or ERK inhibitor treatment. Moreover, a ROCK inhibitor showed augmented melanoma cell death upon BRAF or ERK inhibition. Authors claimed that ROCK1 should be explored as a target in combination with current BRAF mutant melanoma therapies.

This is an interesting study that potentially uncovers a new target for BRAF mutant melanoma treatment. However, some points need to be addressed to improve the manuscript and to support the conclusions.

1. *Phosphoproteomic studies are very variable in terms of phosphopeptides recovery after SCX processing and therefore technical replicates are crucial to support the relevance of the findings. Although authors based their results in 3 biological replicates, it would strengthen the study if the key phospho-proteome changes were validated by Western blot.*

We have now validated phospho-RPS6 Ser235/236 on western blot (Figure 3B). Furthermore, phospho-ERK1/2 is down-regulated both on both western blot (Figure 3A) and mass spectrometry analysis (Table E1).

2. *MS/MS data analysis against the human Uniprot database renders a probabilistic identification of the proteins. Therefore, authors should also validate more total proteins to support the findings. The only protein validated in the manuscript is ROCK1.*

We have now validated additional proteins by western blot and confirmed that calveolin-1, integrin beta1 and NRAS are up-regulated upon three days of PLX4720 treatment (Figure 3C).

3. *The clinical importance of this finding is not clear. The study would be strengthened if the findings were validated by IHC stain of ROCK1 in melanoma samples with BRAF mutation.*

This is an interesting point raised by the reviewer. In our proteomic dataset we found that ROCK1 regulators are changed upon BRAF inhibition. As ROCK1 activity, rather than protein level, is altered following PLX4720 treatment, testing ROCK1 protein level by IHC staining would not add to the manuscript. Measuring ROCK1 activity in clinical samples is not yet possible unfortunately.

4. *The study would be strengthened by in vivo validation using a combination of ROCK1 and BRAF inhibitors to show that their findings hold true in a clinically relevant setting.*

While we agree with the referee that in vivo validation would certainly strengthen our findings, we feel that the proposed experiment is beyond the scope of this manuscript.

5. *Figure 4B-C and Figure5 A-B, it would be better to move the Y axis to the intersection with (-3) in the X axis. It would also be helpful to show in the figure the change in the IC50 value achieved with each treatment.*

We thank the reviewer for this comment and have changed the figures accordingly.

2nd Editorial Decision 20 November 2014

Thank you again for submitting your work to Molecular Systems Biology. We have now carefully considered your revised manuscript. I am glad to inform you that we are satisfied with the modifications made and we think that the work is now suitable for publication.

© European Molecular Biology Organization
